# COVID-19 Timeline of Vietnam: Important Milestones Through Four Waves of the Pandemic and Lesson Learned

**DOI:** 10.3389/fpubh.2021.709067

**Published:** 2021-11-24

**Authors:** Le Huu Nhat Minh, Nguyen Khoi Quan, Tran Nhat Le, Phan Nguyen Quoc Khanh, Nguyen Tien Huy

**Affiliations:** ^1^Faculty of Medicine, University of Medicine and Pharmacy at Ho Chi Minh City, Ho Chi Minh, Vietnam; ^2^Hue University of Medicine and Pharmacy, Hue, Vietnam; ^3^Centre for Tropical Medicine and Global Health, Oxford University Clinical Research Unit, Ho Chi Minh, Vietnam; ^4^School of Tropical Medicine and Global Health, Nagasaki University, Nagasaki, Japan

**Keywords:** COVID-19, SARS-CoV-2, Vietnam, policy, severe acute respiratory syndrome, vaccines, contact tracing, quarantine

Among countries that have suffered the COVID-19 pandemic, Vietnam has successfully prevented the transmission since the early days with no deaths for months. However, the latest wave of the COVID-19 pandemic (the fourth wave) has been damaging Vietnam since then. The case-fatality ratio (CFR) of Vietnam has increased rapidly to a higher number than the average of the world since then. In this spotlight, we do a brief review to summarize the important milestones that Vietnam has gone through and then discuss the important lessons that we have learned from the fight against the COVID-19 pandemic.

According to the Ministry of Health (MOH), Vietnam has suffered four waves of the COVID-19 pandemic (Figure 1). The first wave of the COVID-19 started in Vietnam (lasts for 85 days, from January 23 to April 16, 2020, included 100 community cases) with the original virus strain from Wuhan (China), and no deaths were reported.

**Figure 1:**
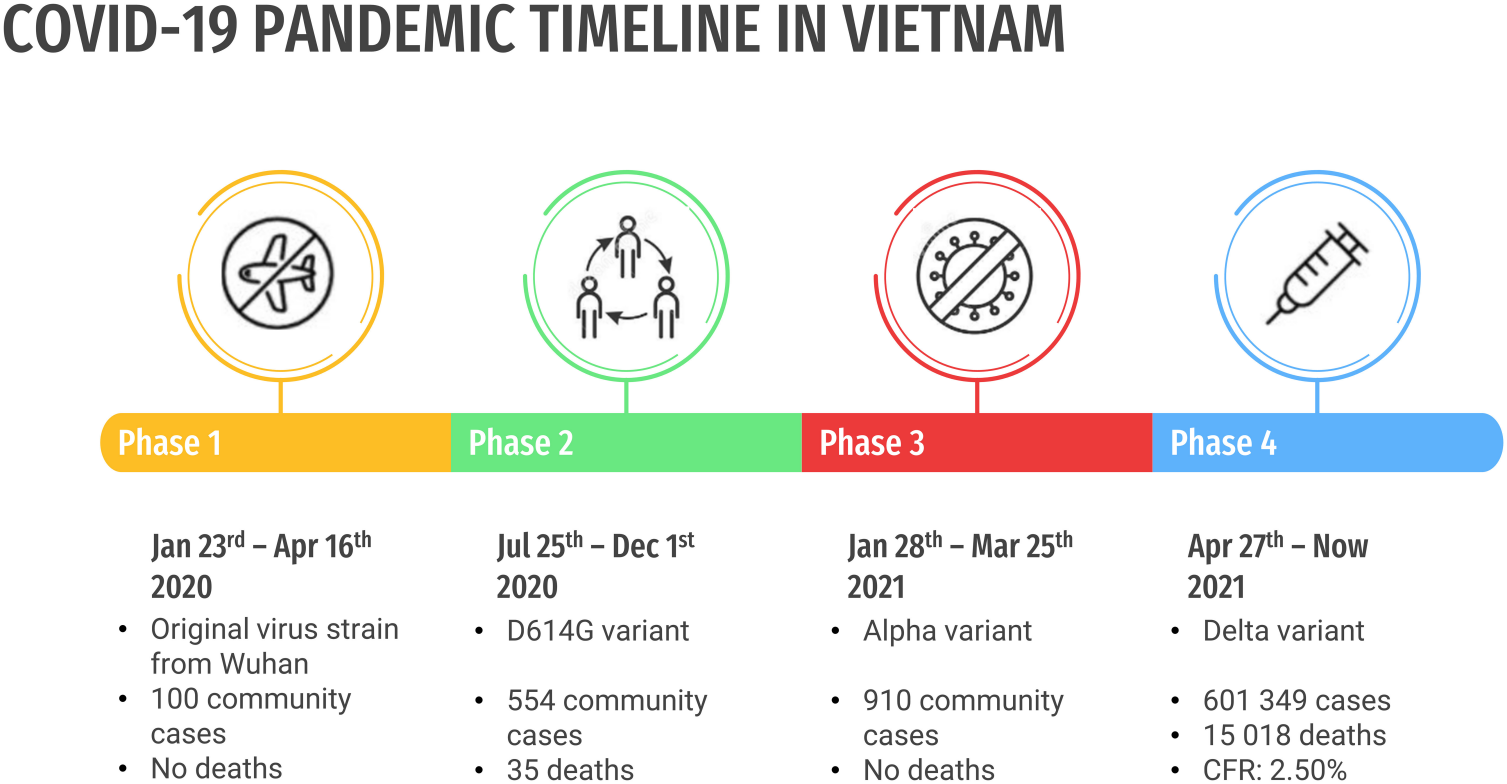
Four waves of the COVID-19 pandemic of Vietnam.

The second wave of the COVID-19 (lasts for 129 days, from July 25 to December 1, 2020, included 554 community cases) started in Da Nang with the same virus strain. A large number of community cases have resulted in 35 deaths in vulnerable patients, who are old and/or have comorbidities. However, the pandemic was successfully controlled.

The third wave of the COVID-19 (lasts for 57 days, from January 28 to March 25, 2021, included 910 community cases) occurred in Hai Duong with the new British variant. Although there were numerous infected people, most of them are young and healthy. Therefore, there is a scarce number of severe cases and no deaths were reported (1–3).

The fourth wave of the COVID-19 pandemic (from April 27, 2021, to now) has dramatically changed the situation, mainly related to the Indian variant (Delta variant). This wave was confirmed as the most complicated and dangerous with most deaths recorded. It has spread not only in hospitals, where many people are seriously ill with many underlying diseases but also in the local communities and large industrial zones. Thousands of community cases have put a massive burden on the whole system including healthcare and contact tracing. As of September 12, 2021, 6,01,349 cases and 15,018 deaths were reported in Vietnam. The CFR was 2.50%, higher than the average number of 2.06% globally (4–6).

We have drawn five lessons called “5Ps strategy” that should be adapted to control and/or prevent the COVID-19 pandemic. These lessons could be useful for the other potential global pandemics that will happen in the future, which included:

1. Prepare a well-designed, sustainable preventive healthcare system from the grassroots level to be ready in case of an emergency.
2. Control the potential waves of the COVID-19 pandemic early when new cases were detected with a combination of contact tracing, isolation, and quarantine.
3. Prevent both the COVID-19 and the non-COVID-19 deaths.
4. Prioritizing the use of the COVID-19 vaccine.
5. “Publish with care”—misinformation on social media leads to harmful consequences.

First, the Vietnam authority has prepared a potent preventive healthcare system from the grassroots level with valuable lessons from the previous respiratory epidemics including SARS (2002–2003) and avian flu (2009) (7, 8). The Vietnamese healthcare system can be classified into two main routes: central and local routes. Each province, or two bordering provinces, have facilities belonging to both the central and local routes. At the basic level, a massive number of “rapid response teams” were founded with members recruited from the local stakeholders (2). Universities and colleges of medicine have organized training courses that aim to equip their students with fundamental knowledge and skills to prevent and control the COVID-19. The students then can participate in the rapid response teams to provide a continuous backup force whenever a pandemic occurs. The force provided enormous local human resources, responsible for tracking all the contacts when new cases are detected, and disseminating necessary healthcare information and policies to the community.

Second, to control the COVID-19 pandemic, we must suppress the potential waves as soon as possible. This strategy can be applied in combination with several measures: detect new cases early, fast tracing and zoning areas of substantial/high transmission, and then applying isolation or quarantine. Residents have to send their health declaration online daily and self-declaration is compulsory in public high-risk areas such as hospitals, schools, or theaters. When a new case was detected, the patients (who are F0) would undergo isolation at once and must indicate their close contact (within a distance of 2 m). The individuals must comply with at least 14 days of isolation and need three negative PCR tests—one at the beginning and two at the end of isolation—to be discharged. Then, the local rapid response teams will thoroughly initiate contact tracing with the F0 following the case investigation prioritization hierarchy. All the close contacts of F0 (called F1) must be taken PCR test immediately and if it is negative, they must comply with 14 days centralized quarantine, later increased to 21 days due to the emergence of the new variants. Individuals who are under isolation need at least three negative PCR tests—one at the beginning and two at the end of quarantine—to be discharged. Then F2—people who closely contacted F1—must also be quarantined in the accommodation facilities. Similarly, F3, F4, and F5—people who closely contacted F2, F3, and F4, respectively—must have a 14-day self-quarantine at home. The entire process is operated and managed by the MOH (4, 9). Encouraging people to use self-testing at home (with COVID-19 rapid testing kit) was also a game-changer for the strategy. This test helped to detect new cases early, quickly, and accurately, especially asymptomatic cases, who can be highly transmissive. It was also easy to use and not expensive for most people.

Third, we need to prevent both the COVID-19 and the non-COVID-19 deaths. The non-COVID-19 deaths included those who were: (1) dead of lack of access to the medical interventions with curable diseases or (2) dead earlier than would be expected of an incurable disease. These non-COVID-19 deaths may also reflect an overwhelmed health system (10). We suggest a three-level model of management to solve this obstacle, which simply classified patients by the severity of the clinical manifestations. Level 1 facilities covered all the asymptomatic or mildly symptomatic patients with medical support from the doctors. Medications should be readily packaged to deliver to these patients including antipyretics and vitamin supplements. Patients with low risk with modest symptoms can be self-managed at home after being examined and prescribed by the doctors with transparent guidelines if the symptoms get worse. The process can also be done via telephone or other mobile platforms. A large number of the COVID-19 cases were mild/moderate and self-limited; only a scarce number with specific risk factors became severe that required hospitalization or intensive care (11–13). We should not admit all the symptomatic patients to prevent system overwhelming in the later phase. Level 2 hospitals admitted moderate/severe symptomatic cases, who were at high risk of worsening. These COVID-19 vulnerable populations include people who are old (aged over 65 years); having some specific medical conditions (cancer, obesity, hypertension, diabetes mellitus, etc.); or who have suffered long-standing systemic health and social inequities (ethnic minorities, disabilities, and slum dwellers) (11, 14). Additional advanced medications can be used such as corticosteroids, antithrombotic therapy, or oxygenation. Level 3 consists of tertiary hospitals with modern equipment and professional doctors, usually the intensive care units (ICUs). This level is in charge of providing supportive treatment with advanced medications and equipment for a small number of severe/critical cases only. Besides the COVID-19, maintaining treatment of other comorbidities should be reminded for a comprehensive care program. Medical mobilization and social mobilization are needed to establish more homecare services and field hospitals, if new cases were rising dramatically out of control. The model helps to allocate the resources appropriately, reducing the burden for the hospitals and ICUs, which resulted in better patient care and a possibly lower number of COVID-19 deaths.

Fourth, prioritizing the use of the COVID-19 vaccine is crucial. As the SARS-CoV-2 can hardly be eradicated, all the residents should have an adaptive immunity against the COVID-19 to begin “a new normal” state. There are two ways to achieve it through natural infection with SARS-CoV-2 or vaccination. Natural infections are proven to be effective to activate long-lasting immunity against the COVID-19 and might even be better than vaccine induced. With the global shortage of vaccines, it should be reserved for the COVID-19 vulnerable populations aforementioned, who are more likely to present severe or critical disease, if infected and who are at high risk of exposure with COVID-19 (i.e., frontline healthcare workers). After covering the people, vaccines can be rolled out progressively for middle- and then low-risk populations. Besides, most of the COVID-19 cases suffered modest symptoms with no complications. Despite being panic because of the daily reported cases, we should take a look at the CFR or total deaths per 1,00,000 population instead to have an exact view of the devastation of the COVID-19 pandemic. “It is as easy for the strong man to be strong, as it is for the weak man to be weak”—R W Emerson. This strategy with the prioritizing groups is quite simple and has proven to be effective in many countries. Israel was a symbolic illustration. By having fewer groups and a lower threshold for age, they made it easier for logistics, reduced roll-out delays, and burden on hospitals. They have controlled the COVID-19 pandemic with CFR = 0.6% and 81.05 deaths per 1,00,000 population, which is quite low in comparison to the average of the world (5, 15). Vietnam has been negotiating for more dosages through the COVAX initiative. With respect to the national vaccine production, four national corporations are participating in the research and development of vaccines. One of them, Nanogen (Nanocovax) finished phase I and II and has recruited more than 10,000 participants for the phase III clinical trial (ClinicalTrials.gov number, NCT04683484). The results of safety and immunogenicity were submitted and under review.

Last but not least, we suggest “publish with care.” The Covid-19 pandemic and intervention measures against it have challenged the mental health status of people, made them feel stressed, overwhelmed, and ultimately led to negative emotions. Social media affected how people perceive the real world. Misinformation on social media can bend reality and lead to unpredicted consequences. It can vary from the contents of the pandemic situations, the COVID-19 treatments to vaccination, or antivaccine. For example, according to the COVID-19 pandemic situation, thousands of new cases were daily reported and published in the media. As we discussed, most of the cases suffered no more than mild/moderate symptoms. We should instead focus on the severe or death rate. However, the number of thousands of new cases was frightening to many people. Images of shoplifting at supermarkets; chaotic crowds jostling for food, medicine, and essentials; or the scenario of cluttered, empty shelves contrast with hundreds of people awaiting payment have become quite popular. The situation reflected excessive panic of the people with the imagination of widespread, uncontrolled disease. We called it the “COVID-19 crowd syndrome.” Nothing is scarier than a crowd that is full of panic and fear.

Besides “the 5Ps strategy,” other strict methods with heavy penalties also lay the foundation for disease control and prevention in Vietnam. “The 5Ks” of actions (five actions which started with the letter “K” in Vietnamese) have been announced, which were both effective and easy to approach for the majority. “The 5Ks” consist of the five crucial methods: face mask, disinfection, social distancing, stop gathering, and practice health declaration that helps to reduce transmission and to adapt to a “new normal” state. The phone call waiting for the ringtone was replaced by the voice reminders regarding antipandemic regulations (1). On April 1, 2020, when the transmission was getting more serious in the first stages, the government imposed a national wide social distancing for 15 days (4). In the later phases, some lockdowns were locally laid out in some COVID-19 hotspots including major cities with numerous confirmed cases and provinces having community cases (domestic cases with unknown sources of infection). This smooth transition in policy reduced the negative consequences of the lockdown on the nationwide level. Citizens have shown their high vaccine acceptance (98%) (16) and high adherence to personal and community preventive methods, which also help to prevent the COVID-19 from further spreading (17).

## Author Contributions

LM, NKQ, and TNL have provided significant contributions to the conception and the design of the manuscript and image credit. All writers have assisted in drafting the manuscript. NTH revised it critically. All authors read and accepted the final version of the manuscript.

## Conflict of Interest

The authors declare that the research was conducted in the absence of any commercial or financial relationships that could be construed as a potential conflict of interest.

## Publisher's Note

All claims expressed in this article are solely those of the authors and do not necessarily represent those of their affiliated organizations, or those of the publisher, the editors and the reviewers. Any product that may be evaluated in this article, or claim that may be made by its manufacturer, is not guaranteed or endorsed by the publisher.
